# Screening for stowaway exotic arthropods imported into Europe via aircraft: A cross-sectional analysis

**DOI:** 10.1016/j.nmni.2025.101660

**Published:** 2025-11-12

**Authors:** Nadja Hedrich, Michèle Bandoly, Juliane K. Fischer, Patricia Schlagenhauf

**Affiliations:** a University of Zurich, Epidemiology, Biostatistics and Prevention Institute, Zurich, Switzerland; b Umweltbundesamt, Berlin, Germany; c WHO Collaborating Centre for Travellers' Health, Department of Global and Public Health, MilMedBiol Competence Centre, Zürich, Switzerland

**Keywords:** Mosquitoes, Aircraft, Airports, Arthropods, Epidemiological monitoring, Travel

## Abstract

**Background:**

International air mobility is increasing. With a changing climate in Europe, and the associated expansion of regions suitable for exotic vector habitats including mosquitoes, it is crucial to investigate possible routes for their transport over long distances. This study investigated whether exotic vectors are introduced to Europe via air traffic.

**Methods:**

At Zürich Airport, passenger cabins of selected aircraft were vacuumed after passengers had disembarked. The samples were examined microscopically, the findings identified, and the arthropod species morphologically determined. The take-off airports were selected based on the occurrence of exotic mosquitoes of interest.

**Results:**

A total of 37 aircraft were sampled between 2021 and 2023. No mosquitoes were detected in any of the samples. However, 12 flights, one third of all flights screened, showed the presence of different arthropods. These included beetles (*Coleoptera*), ants (*Hymenoptera)*, and a fly (*Diptera*). None of the insects found were vectors of importance for human diseases, and there was no correlation found between presence of insects on the aircraft and the aircraft size or jetty type at destination.

**Conclusions:**

The results of this study indicate that the risk of mosquitoes being introduced into the passenger cabin of an airplane is low. However, the survival of mosquitoes introduced from tropical regions is favoured by warming climatic conditions in Europe. We suggest that further studies using traps at airports and in aircraft cargo holds are warranted. Constant surveillance and random sampling of aircraft for arthropods, particularly at high volume hubs, are recommended.

## 1 Introduction

Air travel is increasing and the IATA (International Air Transport Association) reported a record high of global air passengers in 2024 with total air traffic increasing by 10.4 % compared to 2023 [1]. With increased mobility there is a heightened risk to public health in terms of the dissemination of infection and the spread of disease vectors. International air transportation can result in the carriage of non-native disease vectors and this is considered a significant threat particularly to island populations and to urban regions with international airports [2]. Exotic mosquito species can board an aircraft in a country of origin, travel thousands of kilometres in the cargo or passenger cabin and leave the aircraft after landing [3,4]. “Stowaway” mosquitoes have been documented in airport hubs and around airports [[5], [6], [7]]. In 2010 and 2011, ten exotic mosquitoes were collected from 38 airplanes returning from Africa in Schiphol airport, the Netherlands [8].

These mosquitoes can be vectors of infectious diseases such as malaria (*Anopheles gambiae*), yellow fever (*Aedes aegypti)*, dengue, Zika, chikungunya (*Aedes* spp.) and West Nile fever (mainly *Culex* mosquitoes). Stowaway arthropods are not necessarily infected with arboviruses or parasites. However, if they are infected, they pose a risk to naïve human populations. Even if uninfected, these vectors may become established in the country of the destination airport. Therefore, stowaway mosquitoes pose a double pronged risk in that they can actively transmit infection in the surroundings of the airport and/or can become established in regions where they were not previously present.

This can also be seen when examining the increasing cases of arthropod-borne viruses in Europe. Outbreaks of exotic mosquito-borne infections have been seen in recent years, notably dengue in Madeira, and chikungunya in Italy, driven by the establishment of exotic mosquito populations in these countries [9,10]. In addition, cases of airport malaria, which is the infection of individuals working or residing close to international airports with malaria, occur every year in Europe. These cases are also increasing, with a recent cluster of three cases seen at the Frankfurt airport in 2022 [11]. Airport malaria is particularly dangerous, as the diagnosis is often delayed due to a lack of travel history, resulting in more serious outcomes or death [12].

One method of preventing the importation of exotic arthropods is aircraft disinsection. This involves spraying insecticides such as permethrin and/or d-phenothrin and 1R-trans-phenothrin within the aircraft [13]. There are several recommended spraying methods including both long and short-term treatments, and treatments at different time points on the trip (pre-embarkation, pre-departure, or on-arrival) [5,13]. A recent evidence review from the WHO outlines these disinsection methods and reviews available evidence [4].

The threat of stowaway mosquitoes is likely to increase with the increase in the volume of international aviation and the globalisation and diversification of flight patterns. In addition, climate change may favour the establishment of exotic arthropod vectors in Europe. Mosquitoes need warmer and wetter conditions to survive and become established in an area, with optimal temperatures of around 20–34 °C depending on the species [14]. Forecasts of future climate conditions show Europe, especially northern Europe becoming warmer and wetter, and, in the next 100 years, over a billion more people may be exposed to exotic mosquitoes [14]. This may imply consequences for public health in these countries.

The goal of this study was to evaluate the risk of exotic arthropods, including mosquitoes, stowing away on aircraft arriving in central Europe from destinations in tropical and subtropical areas.

## 2 Methods

### 2.1 Protocol for passenger cabin sampling

A Pre-Flight

Before taking the sample, data from the flight was recorded, including flight number, type of aircraft, country and airport of origin, insecticide spraying status of the aircraft, date, and time of arrival. Three different aircraft models were sampled with the vacuum method: These aircraft types were: A319, A320, and A340s. All were Airbus models of different sizes; the A319 can accommodate up to 156 passengers, the A320 up to 186, and the A340 up to 380 passengers. The A340 aircraft have a longer range, and service farther away destinations, such as Costa Rica and Sri Lanka. Also recorded was whether the planes were connected to the main airport building with a closed jetty at the destination, or gangway open to the environment, which may also play a role in how easily arthropods can enter the passenger cabins while the aircraft is waiting at the destination.

B Sampling procedure

After the flight arrived and all the passengers had disembarked, and before the cleaning crews arrived, sampling and searching for arthropods started. Using a handheld cordless vacuum, the cabin of the aircraft was systematically vacuumed along the sides of the aircraft from the left-hand side down to the end of the aircraft and then back up on the right-hand side. The whole aircraft was swept, including any premium seating sections when these were present. The sweeps took an average of 15 min per aircraft.

**Fig. 1:**
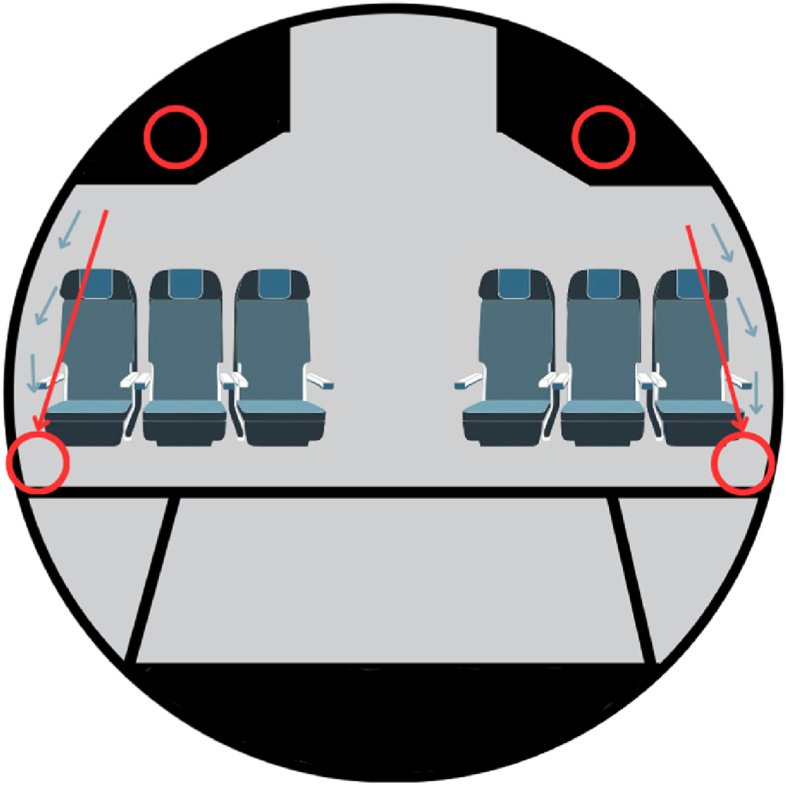
Cross sectional schematic of an aircraft showing airflow patterns (blue arrows), expected trajectory of mosquitoes (red arrows) and location of sample collections (red circles).

The justification for this is due to a combination of the airflow of an aircraft, where the mosquito vector would likely be pushed along the sides and dead mosquitoes are more likely to both accumulate along the edges of the cabin, but also to be likely better preserved (Fig. 1). This protocol has been previously validated also using a handheld chargeable vacuum device [15]. In addition to the sides of the aircraft, the overhead baggage compartments were visually examined, and anything seen was vacuumed, and the whole cabin was visually scanned for living mosquitoes, which would also have been captured.

The vacuum cleaner used was the Distianert 120W, 7000 PA, cableless hand vacuum, chosen because of its quick charging time, cordless use, and useful attachments to better access the corners and edges of the aircraft.

C Sample storage/Analysis

**Fig. 2:**
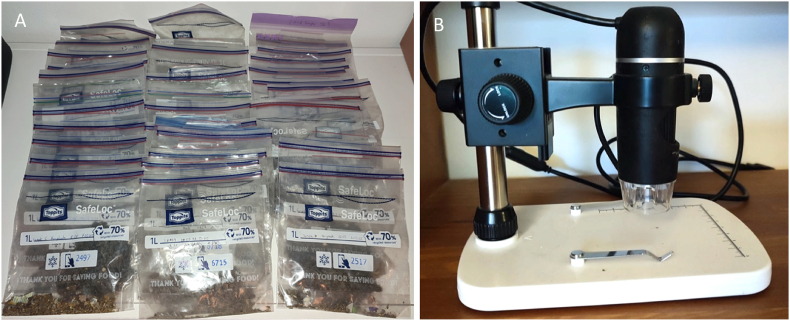
A. Samples Taken from Aircraft. B. MicroCapture Pro Digital Microscope was used for sample analysis.

After the samples were taken, they were transferred from the vacuum to a sealed bag with the date and flight number recorded and stored until they were examined for mosquitoes and other arthropods (Fig. 2 A).

The individual samples were then examined under a microscope in a glass dish to look for whole, or fragments of mosquitoes or other insects (Fig. 2 B). Each sample was examined in aliquots of 1–2 g.

Any mosquitoes or arthropods or fragments found were morphologically typed to genus or species if possible.

Maps were generated using the statistical programme R [16].

## 3 Results

**Fig. 3:**
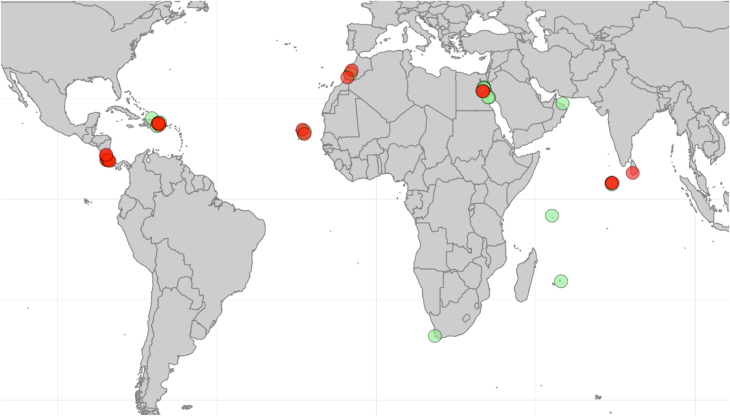
Locations of all flights sampled (n = 37). Locations in red had arthropods found on board, locations in green had no arthropods found. Some locations had more than one flight sampled.

In total, 37 flights to Zürich airport were sampled, primarily in the periods of October to December 2021, and May 2023 (Table 1). Flights from 17 locations were examined, including origins in the Caribbean (Costa Rica and Dominican Republic), South Asia (Sri Lanka, Maldives), North Africa (Egypt and Morocco), and Island nations off Africa (Mauritius, Seychelles, Cabo Verde) (Fig. 3). One cargo trap was placed in the hold on a flight to Cape Town, South Africa, and back. From these 37 flights, no mosquitoes or mosquito fragments were identified; however, 12 samples had arthropods or arthropod fragments present (Table 1). No pattern was seen with respect to the presence of arthropods in the samples, and presence or absence of an aircraft jetty, or size of the aircraft. A chi-squared test examining jetty presence and the detection of arthropods on board showed no significance under the Yates correction (p = 0.11).

**Table 1:** Results from cabin sampling for insects at Zürich airport (n = 37).

Date	Country of Origin	Plane type	Jetty	Findings
06.10.21	Egypt	A319	No	0
08.10.21	South Africa	A343	NA	0^a^, ^b^
20.10.21	Egypt	A319	No	0
03.11.21	Egypt	A319	No	0
08.10.21	Egypt	A319	No	0
10.03.23	Egypt	A319	No	0
31.03.23	Egypt	A319	No	0
04.05.23	Costa Rica	A340	Yes	Unknown
04.05.23	Dominican Republic	A340	Yes	0
04.05.23	Egypt	A320	No	0
05.05.23	Egypt	A320	No	0
05.05.23	Sri Lanka - Maldives	A340	*Yes*	*Coleoptera*
06.05.23	Costa Rica	A340	Yes	*Formicidae*
06.05.23	Oman	A340	Yes	0
07.05.23	Egypt	A320	Yes	Unknown
07.05.23	Dominican Republic	A340	No	*Coleoptera*
08.05.23	Mauritius-Seychelles^b^	A340	Yes	0
09.05.23	Egypt	A320	Yes	0
09.05.23	Cabo Verde	A320	No	*Diptera*
10.05.23	Morocco	A320	Yes	*Coleoptera*
11.05.23	Dominican Republic	A340	No	0
11.05.23	Costa Rica	A340	Yes	Unknown
11.05.23	Egypt	A320	No	0
13.05.23	Morocco	A320	Yes	0
14.05.23	Dominican Republic	A340	No	0
14.05.23	Maldives	A340	No	0
18.05.23	Dominican Republic	A340	No	0
18.05.23	Costa Rica	A340	Yes	0
16.05.23	Cabo Verde	A320	No	0
17.05.23	Egypt	A320	Yes	*Formicidae/Rhophalidae*
21.05.23	Dominican Republic	A340	No	0
21.05.23	Maldives	A340	No	Unknown
23.05.23	Cabo Verde	A320	No	0
25.05.23	Dominican Republic	A340	*No*	*Araneae*
28.05.23	Dominican Republic	A340	*No*	*Formicidae*
28.05.23	Maldives	A340	No	0
30.05.23	Cabo Verde	A320	No	0

a Flight was sampled using a Biogents Pro trap.

b Flight from the Seychelles was sprayed with insecticide, the others were not.

**Fig. 4:**
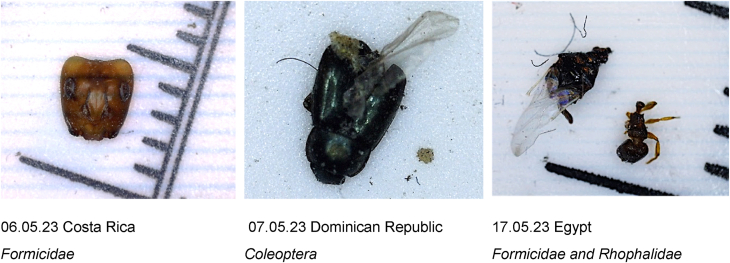
Examples of arthropods and fragments found in collected samples from aircraft cabins at Zürich airport.

Arthropods identified included several ants (*Formicidae)*, beetles (*Coleoptera*), as well as a spider (*Araneae*) and fly (*Diptera*) (Fig. 4). None of the samples that were able to be identified contained an insect with importance as a vector of human disease.

## 4 Discussion

This research project addresses an important aspect of public health – the potential introduction of stowaway arthropods to Europe through air travel. This approach aims to provide an understanding of the risk of exotic mosquitoes arriving on flights. The focus on flights arriving from tropical areas and the selection of flights via Zürich Airport, are considered representative of flight traffic to Switzerland and Germany and Central Europe. The departure airports were chosen based on the presence of potential vectors, and vector-borne diseases.

The use of mosquito surveillance programs at international airports (Narita Airport, Tokyo, Japan and Schiphol Airport, Amsterdam, the Netherlands) has already provided evidence of mosquito transport on aircraft [[17], [18], [19]]. We built upon this foundation by directly sampling and analysing passenger compartments for mosquitoes using a sampling method of vacuuming along the sides of the passenger cabin and visually inspecting the overhead luggage compartments.

The absence of mosquitoes on the sampled flights to Zürich airport suggests that stowaway mosquitoes occur rarely, but it does not definitively rule out the potential risk of stowaway mosquitoes arriving on flights or at other airports. Additionally, the presence of fragments of different arthropods on 12 flights, one third of all flights sampled, highlights the broader issue of arthropod transport, not limited to mosquitoes. Therefore, different methods of arthropod introduction on aircraft should also be considered, as flying insects such as mosquitoes, flies, and beetles may enter through the open doors when an aircraft is not anchored by jetty at the origin airport, and others such as ants or non-flying beetles may achieve ingress through transportation on the clothing or luggage of the passengers themselves. These different entry ways should be examined separately, as there may be different methods of prevention recommended such as “wind curtains” [5]. However, when examining the association of arthropods found on aircraft and the presence of a jetty, no significant association was found. Other arthropods, which could potentially carry diseases or be nuisance pests, might also pose a risk. The implications of these arthropods would need further exploration to assess their significance in terms of public health risk. Certain non-mosquito arthropods that may be considered a public health risk would include other vectors of human disease such as ticks, sandflies, lice, and fleas, or other pests such as bedbugs. Future studies may also consider using molecular methods in addition to morphological identification for identification of insect fragments, or to examine whether any imported arthropods are carrying viruses.

Other studies examining aircraft for the presence of mosquitoes had varied results, with a study conducted at Schiphol Airport between 2010 and 2011, finding ten out of 38 aircraft with exotic mosquitoes present, and a study at the Frankfurt airport finding only one mosquito thorax in 43 aircraft, and reporting no other arthropods [8,15]. However, in comparison to the large number of flights arriving at European airports daily, these sample sizes may be a limitation. In addition, various other factors could contribute to the variance in mosquito presence observed across these studies. An important strength of our study is that different models of aircraft were included, a mixture of jetty, and open-air status at the departure airport, and a variety of destinations, all with populations of exotic mosquitoes. A difference in airline policy may account for some of these differences seen, including how well the aircraft are cleaned between flights, or how long the doors remain open at the destination. In addition, future studies examining the cargo compartments of aircraft would provide valuable information on another possibility of mosquito introduction, but this is constrained by airport security issues, Models using air traffic volume, destination vector epidemiology and seasonality may predict the risk of stowaway mosquitoes and arthropods on air routes and may be a basis for the implementation and timing of control measures such as aircraft disinsection [4]. Due to changing climatic conditions and the widening of geographic areas suitable for vectors of human infections, there is a need for continuous risk assessment evaluations. Such a monitoring would be a useful approach to the surveillance of the movement of vectors via aviation. A recent systematic review from WHO identified 8 surveillance studies in Europe that showed presence of exotic mosquitoes at airports [4]. The WHO advocates an “*integrated vector management***”** which is a rational decision-making process to optimize (the use of resources for) vector control [13]. As there is a strong risk that vectors will enter aircraft at airports with high vector populations, airlines should encourage airport management to organize vector surveillance and control within and in at least a 400-m perimeter of the airport in accordance with Annex 5 of the International Health Regulations [20]. The reduction of vector populations in and around airports will reduce the risk of arthropods entering aircraft.

## 5 Conclusion

In conclusion, the research project contributes to our understanding of the risk of stowaway vectors, particularly mosquitoes, on flights to Europe. The absence of mosquitoes in the cabins of the sampled flights suggests a relatively low immediate risk, but the presence of other arthropods warrants further investigation. However, stowaway mosquitoes have been reported, and further assessment of the magnitude of the phenomenon is needed. We suggest that studies using traps at airports and in aircraft cargo holds are conducted. Constant surveillance and random sampling of aircraft particularly at high volume hubs are recommended. The findings contribute to the ongoing discussion on the necessity and effectiveness of disinsection measures, advocating for a more systematic and standardized decision-making process based on comprehensive risk assessments.

## CRediT authorship contribution statement

**Nadja Hedrich:** Writing – original draft, Project administration, Methodology, Investigation, Formal analysis, Data curation, Conceptualization. **Michèle Bandoly:** Writing – review & editing, Conceptualization. **Juliane K. Fischer:** Writing – review & editing, Conceptualization. **Patricia Schlagenhauf:** Writing – review & editing, Validation, Supervision, Methodology, Funding acquisition, Conceptualization, Writing – original draft.

## Funding

This project was funded through the German Federal Ministry for the Environment, Nature Conservation, Nuclear Safety and Consumer Protection (BMUV), through the Federal Environment Agency (UBA), grant number FKZ 3720 48 402.

## Declaration of competing interest

The authors declare the following financial interests/personal relationships which may be considered as potential competing interests: Prof. Dr. Patricia Schlagenhauf, Dr. Nadja Hedrich report financial support was provided by the German Federal Ministry for the Environment, Nature Conservation, Nuclear Safety and Consumer Protection (BMUV), through the Federal Environment Agency (UBA), grant number FKZ 3720 48 402. If there are other authors, they declare that they have no known competing financial interests or personal relationships that could have appeared to influence the work reported in this paper.
